# Commentary: Comparison of Outcomes in Immediate Implant-Based Breast Reconstruction: Acellular Dermal Matrix versus Inferior Dermal Flap

**DOI:** 10.1055/s-0043-1777251

**Published:** 2024-02-22

**Authors:** Luís Mata Ribeiro, Rita P. Meireles, Irís M. Brito, Patrícia M. Costa, Marco A. Rebelo, Rui F. Barbosa, Miguel P. Choupina, Carlos J. Pinho, Matilde P. Ribeiro

**Affiliations:** 1 Plastic and Reconstructive Surgery Department, Centro Hospitalar Universitário Lisboa Central, Hospital São José, Lisbon, Portugal; 2 Plastic and Reconstructive Surgery Department, Centro Hospitalar e Universitário de Coimbra, Coimbra, Portugal; 3 Plastic and Reconstructive Surgery Department, Instituto Português de Oncologia do Porto, Porto, Portugal

Comparison of Outcomes in Immediate Implant-Based Breast Reconstruction: Acellular Dermal Matrix versus Inferior Dermal Flap

Implant-based breast reconstruction has evolved tremendously in the last decades, mainly due to the development of new products and techniques that make the procedure safer and more reliable. The purpose of this study was to compare the outcomes in immediate one-stage breast reconstruction between acellular dermal matrix (ADM) and inferior dermal flap (IDF). We conducted a retrospective comparative study of patients submitted to immediate breast reconstructions with an anatomical implant and ADM or IDF in a single center between 2016 and 2018. Outcomes evaluated included major complications, early complications, reinterventions, readmissions, and reconstruction failure. Simple descriptive statistics and univariate analysis were performed. A total of 118 breast reconstructions (85 patients) were included in the analysis. Patients in the IDF group had a higher body mass index (median = 27.0) than patients in the ADM group (median = 24). There were no statistically significant differences among both groups regarding immediate major complication, early complications, readmissions, and reinterventions. There are no significant differences in complications between the ADM and IDF approach to immediate implant breast reconstruction. In patients with higher body mass index and large, ptotic breasts, we recommend an immediate implant reconstruction with IDF.



